# p62/SQSTM1 interacts with vimentin to enhance breast cancer metastasis

**DOI:** 10.1093/carcin/bgx099

**Published:** 2017-09-12

**Authors:** Si-Si Li, Ling-Zhi Xu, Wei Zhou, Shang Yao, Chun-Li Wang, Jiang-Long Xia, He-Fei Wang, Muhammad Kamran, Xiao-Yuan Xue, Lin Dong, Jing Wang, Xu-Dong Ding, Laura Bella, Laurence Bugeon, Jie Xu, Fei-Meng Zheng, Margaret J Dallman, Eric W F Lam, Quentin Liu

**Affiliations:** 1 Cancer Center, Institute of Cancer Stem Cell, Dalian Medical University, Dalian, China; 2 State Key Laboratory of Oncology in South China, Collaborative Innovation Center for Cancer Medicine, Sun Yat-sen University, Guangzhou, China; 3 Department of Oncology, the Second Affiliated Hospital of Dalian Medical University, Dalian, China; 4 Department of Surgery and Cancer, Imperial College London, London W12 0NN, UK; 5 Department of Oncology, the First Affiliated Hospital of Dalian Medical University, Dalian, China; 6 Department of Pathology, the Second Affiliated Hospital of Dalian Medical University, Dalian, China; 7 Department of Life Sciences, Imperial College London, London SW7 2AZ, UK

## Abstract

The signalling adaptor p62 is frequently overexpressed in numerous cancer types. Here, we found that p62 expression was elevated in metastatic breast cancer and its overexpression correlated with reduced metastasis- and relapse-free survival times. Analysis of p62 expression in breast cancer cell lines demonstrated that high p62 expression was associated with the invasive phenotypes of breast cancer. Indeed, silencing p62 expression attenuated the invasive phenotypes of highly metastatic cells, whereas overexpressing p62 promoted the invasion of non-metastatic cells in *in vitro* microfluidic model. Moreover, MDA-MB-231 cells with p62 depletion which were grown in a three-dimensional culture system exhibited a loss of invasive protrusions. Consistently, genetic ablation of p62 suppressed breast cancer metastasis in both zebrafish embryo and immunodeficient mouse models, as well as decreased tumourigenicity *in vivo*. To explore the molecular mechanism by which p62 promotes breast cancer invasion, we performed a co-immunoprecipitation–mass spectrometry analysis and revealed that p62 interacted with vimentin, which mediated the function of p62 in promoting breast cancer invasion. Vimentin protein expression was downregulated upon p62 suppression and upregulated with p62 overexpression in breast cancer cells. Linear regression analysis of clinical breast cancer specimens showed a positive correlation between p62 and vimentin protein expression. Together, our findings provide strong evidence that p62 functions as a tumour metastasis promoter by binding vimentin and promoting its expression. This finding might help to develop novel molecular therapeutic strategies for breast cancer metastasis treatment.

## Teaser

p62 functions as a tumour metastasis promoter through its interaction with vimentin. This finding might help to develop novel molecular therapeutic strategies for breast cancer metastasis treatment.

## Introduction

Cancer metastasis is a complex multi-step cell biological process termed the invasion-metastasis cascade, by which primary tumour cells acquire the invasive ability and then spread to distant organs (1–3). Metastases are responsible for more than 90% of cancer-related deaths in various solid malignancies, including breast cancer (4,5). Despite the early diagnostic methods, surgical resection and adjuvant therapy have improved, metastatic disease is largely incurable (6). Recent studies have shown that the primary cause for cancer cells to drive the invasion-metastasis process is the acquisition of the genetic or epigenetic alterations (7,8). Thus, the exploration of key molecules and mechanisms underlying breast cancer metastasis regulation is urgently needed.

The adaptor protein p62 (also known as SQSTM1) is initially identified as a cytosolic 62kDa protein which can bind to the isolated src homology 2 (SH2) domain of p56^lck^ (9). This multi-domain protein interacts selectively with different signalling intermediaries, such as Raptor (10), Nrf2-binding site on Keap1 (11), ubiquitin and LC3 (12,13), to regulate metabolic reprogramming, antioxidant response and selective autophagy, respectively. Abnormal p62 overexpression has been documented in various neoplasms (14–19), especially in breast cancer (20–22). For example, the scaffold p62 protein cooperates with the Wnt/PCP protein VANGL2 and recruits JNK to VANGL2, which is required for tumour growth in breast cancer (23). In addition, high p62 expression is associated with breast tumours exhibiting the clinicopathological features of aggressive disease, as well as overexpression of EGF receptor, HER2, HER3 and HER4 (21). In triple-negative breast cancers, patients with p62 accumulation exhibit a higher risk of positive lymph node and lymphovascular invasion (22). These findings highlight the potential of p62 as a therapeutic target during cancer progression. However, the detailed mechanism by which p62 mediates cancer cell invasion and metastasis remains largely unknown.

Studies of epithelial malignancies indicate that the acquisition of invasion and metastasis potential by the incipient cancer cell may depend on the transition of a mesenchymal phenotype (24). Vimentin, a Type III intermediate filament, serves as a classical mesenchymal phenotype biomarker (25,26). Furthermore, vimentin protein overexpression positively correlates with cell motility, induction of Epithelial-Mesenchymal Transition, metastatic disease and poor prognosis (27–31). *Vimentin*-deficient (−/−) mice reveal weakened wound healing ability in all stages of growth as a result of the seriously impaired fibroblasts in their capacity to migrate (32,33). In this study, we found that p62 binds to vimentin and regulates vim entin protein level, which in turn contributes to cancer cell invasion and metastasis. Thus, this p62–vimentin interaction may be a promising target and provide new opportunities for therapeutic intervention.

## Materials and methods

### Clinical samples, cell culture and reagents

All breast cancer specimens and adjacent normal tissues were obtained from clinical diagnosed patients with prior patient consent and the approval of the Institutional Clinical Ethics Review Board in the Second Affiliated Hospital of Dalian Medical University. Samples were kept in liquid nitrogen for protein extraction.

The human breast cancer cell lines (MDA-MB-231, SKBR-3, BT549 and MCF7), the immortalized human breast epithelial cell MCF-10A and human embryonic kidney HEK293T cell line were obtained from the American Type Culture Collection. The SUM149 cell line was kindly provided by Prof. Zhi-Min Shao (Deparment of Medical Oncology, Cancer Hospital of Fudan University, Shanghai Medical College, Shanghai, China). All the cell lines were tested and authenticated by the standard short tandem repeat DNA typing methodology before used in this study (34). Each cell line was cultured in its standard medium as recommended by American Type Culture Collection. SUM149 cells were cultured in F-12 Hams (Gibco) supplemented with 5% FBS (Hyclone), 5 μg/ml insulin (Sigma–Aldrich), and 1 μg/ml hydrocortisone (Sigma–Aldrich). The cells were last tested on 7 July 2015.

### Plasmid construction

Full-length of p62 fragment was cloned from human genome cDNA and ligated into pLVX-DsRed-Monomer-N1 vector (Clontech). The primers were as followed: p62: 5′ XhoI, 5′-CCGCTCGAGCGGGCCA CCATGGCGTCGCTCACCGTGAAGGCC; 3′ EcoRI, 5′-CCGGAATTCCGTCA CAACGGCGGGGGATGCTTTGA. Flag-p62 was a gift from Prof. Song-Shu Meng (Department of Cancer Biology, Dalian Medical University, Dalian, China). The short hairpin RNA (shRNA) targeting p62 (1#, 2#) and the non-target shRNA (SHC002) were kindly provided by Dr Zi-Jie Long (Department of Hematology, the Third Affiliated Hospital, Sun Yat-sen University, Guangzhou, China) and packaged for lentivirus particles. The full-length vimentin plasmid was cloned from human genome cDNA and ligated into pcDNA6 vector in a frame with Myc and His tag (Invitrogen). The primers were as followed: vimentin: 5′ KpnI, 5′-GGGGTACCCCGCCACCAT GTCCACCAGGTCCGTGTCC; 3′ EcoRI, 5′-CCGGAATTCGGTTCAAGGTCATCG TGATGCTGAGAAG. The constructs pLVX-GST-p62 and pLVX-GST-vimentin were generated by PCR and confirmed by sequencing. Flag-Ub was a gift from Dr Muhammad Kamran (Department of Cancer Biology, Dalian Medical University). The shRNA constructs targeting Vimentin and referring to the sequence is: Forward, CCGGCTCTGGTTGATACCCACTCAACTCGAGTT GAGTGGGTATCAACCAGAGTTTTTG; Reverse, AATTCAAAAACTCTGGTTG ATACCCACTCAACTCGAGTTGAGTGGGTATCAACCAGAG. The above fragment was cloned into pLKO-Tet-On-shNC vector.

### RNAi and transfection

Transient RNAi transfection was carried out as previously described (35). Two different target siRNA sequences of p62 were obtained from GenePharma Co. Ltd (1#, 5′-GUGACGAGGAAUUGACAAUTT; 2#, 5′-GGAGUCGGAUAACUGUUCATT). The negative control siRNA was 5′-UUCUCCGAACGUGUCACGUTT. RNAi was transfected into the culture cells using Lipofectamine 2000 Transfection Reagent (Invitrogen) according to the manufacturer’s instructions.

### Lentivirus preparation

HEK293T cells were used for packaging lentivirus with the second generation packaging system plasmid psPAX2 (Addgene) and pMD2.G (Addgene). Lentiviruses were concentrated by ultracentrifugation, and viral titer determined by serial dilutions. For infection with the lentivirus, infected cells were selected with Puromycin (2 μg/ml) (Sigma–Aldrich).

### Microfluidic chips

This microfluidic device consisting of one central chamber and two side channels was adopted in the present study and was modified for certain use (Supplementary Figure 1 is available at *Carcinogenesis* Online). Inlet and outlet ports of the PDMS (poly-dimethyl-siloxane; Silgard 184, Dow Chemical) devices were bored using disposable biopsy punches and the PDMS layer was bonded to a cover glass to create microfluidic channels 80 mm deep with oxygen plasma treatment. These devices were subsequently sterilized by autoclave and dried in oven. Then, Matrigel (BD Biosciences) was mixed with same volume cell culture medium and was injected within the central channel using a 200 µl pipette. The chips were placed in the 10 cm petri dishes which contain 3 ml sterile water and were ready for use after 15 min standing.

### Transwell invasion assay

Cells were placed into 10% matrigel (BD Biosciences) coated membrane in upper chamber (24-well insert, 8 μm, Corning Costar). Medium with 10% FBS was used as an attractant in the lower chamber. After being incubated for 36 h, cells invaded through the membrane were fixed with 75% ethanol and stained with DAPI (1 μg/ml). The stained cell images were captured by microscope (Olympus), and five random fields at ×10 magnification were counted. Results were shown as average from at least three independent experiments. Error bars represented the standard deviation.

### Three-dimensional culture

Three-dimensional culture was carried out as previously described (36). Culture slides (BD BioCoat) were added with 80 μl Matrigel (BD Biosciences) per well and incubated at 37°C for 1 h. Next, cells (2 × 10^3^) mixed with 2% Matrigel were added to each well and refed each 3 days. Finally, we observed the cell morphology under a fluorescence microscope (Olympus).

### Zebrafish embryo xenograft assay

#### Zebrafish maintenance

Zebrafish adult specimens were maintained following the standard guidelines depicted in Nüsslein‐Volhard and Dahm (2002). Zebrafish were kept in a self-recirculating aquarium at an average temperature of 28°C with a 14-h light 10-h dark cycle. Adult specimens were fed twice a day on a diet of Hikari micropellets (Kyorin) and brine shrimp. Zebrafish embryos (1–7 dpf) were kept in a solution composed of system water (chlorine deprived tap and distilled water mixture) with the addition of 0.0003% (v/v) methylene blue (antifungal) at a constant temperature of 28°C.

#### CM-Dil labelling

Cells lines were pre-seeded to obtain a culture with 80% level of confluency on the day of the CM-DiI (Invitrogen) cell labelling. CM-DiI dye was diluted in DMEM without solutes according to the concentrations defined during the DiI-labelling optimization. Cell lines were individually trypsinized and centrifuged to obtain a pellet. Cell pellets were resuspended in 5 μl/ml CM-DiI dilutions. Cells were left to incubate in the dye at 37°C at 10% CO_2_ for 10 min. Following treatment, cells were rinsed once with phosphate-buffered saline and centrifuged twice to remove all excess media before injection. The final pellet was then used for the zebrafish microinjection procedure.

#### Microinjection of human tumour cells

Zebrafish embryos developed in the chorion and have to be dechorionated at 24 hpf before injection. Human tumour cell lines (MCF-7 NSC and MCF-7 p62 siRNA) were pre-labelled with CM-DiI and centrifuged to obtain a dry pellet. The embryos were anaesthetized in a solution containing 0.003% tricane (Sigma) 10 min before injection. Injections were performed on an injection mould composed of 3% agarose in a solution of pre-warmed phosphate-buffered saline (+Mg +Ca) 0.003% tricane, using a 12 mm gage borosilicate pipette fixed on a Narishige microinjector. Embryos were injected in the yolk sac region in proximity of the embryos sub-intestinal vessels with approximately 150 breast cancer cells. Injection were completed within 1 h following which embryos were placed at 28°C in a solution of system water with methylene blue for the duration of the experiment.

Embryos were imaged individually at 3 days post-implantation under a wide-field fluorescent microscope (Olympus CKX41). Tricaine (0.05%) was added to their water to prevent their movement during the live imaging procedure. Pictures were captured with Q Capture-Pro (QImaging). Any alteration of the original picture was performed with the aid of ImageJ. The corrected total cell fluorescence (CTCF) was measured using the formula: CTCF= integrated density − (area of selected cell × mean fluorescence of background readings) for each imaged zebrafish.

#### Analysis of metastasis

Female BALB/C-nu mice (4–6 weeks old) were tail intravenous injected with 1 × 10^6^ cells per mouse. After 8 weeks, lungs were removed and examined macroscopically or detected in paraffin-embedded sections stained with haematoxylin and eosin.

#### Analysis of tumour growth

Female BALB/C-nu mice (4–6 weeks old) were subcutaneously inoculated with equal amounts (1 × 10^6^/100 μl in phosphate-buffered saline containing 50% Matrigel) of single cells. Tumour formation was monitored for 8 weeks. The tumour volumes and weights were determined by the method described previously (34). All animal studies were approved by the Institute Animal Care and Use Committee of Dalian Medical University, and carried out in accordance with established institutional guidelines and approved protocols.

#### RNA extraction, reverse transcription-PCR and real-time quantitative PCR

Total RNA was extracted by using TRIzol reagent (Life technologies). cDNA was generated by using EasyScript One-Step gDNA Removal and cDNA Synthesis SuperMix Kit (TransGen Biotech) according to the manufacturer’s instructions. Real-time quantitative PCR was performed by using the specific SYBR Select Master Mix (Life technologies) in a MX3000p cycler (Stratagene). Changes of mRNA levels were determined by the $2^{-\Delta \Delta C_{T}}$ method using Actin for internal crossing normalization. Detailed primer sequences for qPCR were listed in Supplementary Table 1, available at *Carcinogenesis* Online.

#### Western blot analysis

Samples were lysed on ice in RIPA buffer (50 mM Tris [pH 8.0], 150 mM sodium chloride, 0.5% sodium deoxycholate, 0.1% sodium dodecyl sulfate and 1% NP-40) supplemented with protease inhibitors (1 mM Na3VO4, 1 μg/ml leupeptin and 1 mM PMSF). The protein concentration was determined by the Coomassie brilliant blue dye method. In all, equal amounts of protein per lane were run in 6–15% sodium dodecyl sulfate–polyacrylamide gel electrophoresis gels and subsequently transferred to a nitrocellulose membrane (Millipore) via submerged transfer. After blocking the membrane at room temperature for 1 h, the membrane was incubated overnight at 4°C with various primary antibodies. After incubation with peroxidase-conjugated secondary antibodies (Thermo Scientific) for 1 h at room temperature, the signals were visualized using an enhanced chemiluminescence western blot detection kit (K-12045-D50; Apgbio, Beijing, China) according to the manufacturer’s instructions. The blots were developed using the Bio-Rad Molecular Imager instrument (Bio-Rad). The information of antibodies were listed as follows: Actin (Proteintech, #60008-1), glyceraldehyde 3-phosphate dehydrogenase (KANGCHEN, KC-5G4), p62 (Santa Cruz, sc-28359), p62 (D5E2) (CST, 8025), Vimentin (ALBIOCHEM, #IF01), FLAG-TAG (Protein Tech, 66008-1-Ig), HIS-TAG (Protein Tech, 66005-1-Ig), HIS-TAG (BBI, D110002-0200), Ubiquitin (UBB) (Protein Tech, 60310-1-Ig), LC3B (Sigma–Aldrich, L7543), GOAT ANTI-MOUSE (ABB-Kinase, a25012-1), GOAT ANTI-RABBIT (Protein Tech, sa00001-2), Goat anti-Mouse IgG (HRP conjugated) (Thermo-Pierce, 31430), Goat anti-Rabbit IgG (HRP conjugated) (Thermo-Pierce, 31460).

#### Co-immunoprecipitation analysis, in-gel trypsin digestion, mass spectrometry, immunofluorescence staining and statistical analysis

See Supplementary Materials and Methods, available at *Carcinogenesis* Online.

## Results

### p62 expression is elevated in metastatic breast cancer and its overexpression correlates with poor prognosis

**Figure 1.**
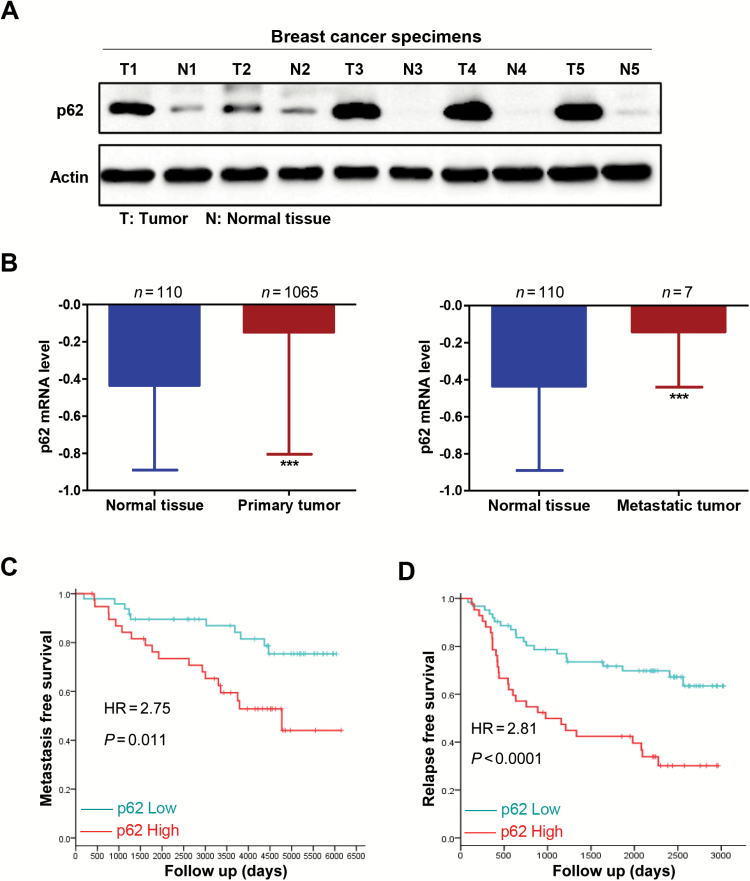
p62 expression is elevated in metastatic breast cancer and its overexpression correlates with poor prognosis. (**A**) p62 protein levels in five pairs of clinical metastatic breast cancer (T) and adjacent normal tissues (N) were subjected to western blot analysis. (**B**) p62 mRNA expression levels in normal tissues and primary tumour (left) or in metastatic tumour tissues (right) were shown by analysis of gene expression value from TCGA. ***P < 0.001, two-tailed Student’s *t*-tests. Error bars represented mean ± standard deviation. (**C**) Kaplan–Meier survival analysis of 87 breast carcinoma specimens from the GEO database (GSE6532) (*P* = 0.011). (**D**) Relapse-free survival times of 104 breast cancer samples from GEO database (GSE42568) by univariate analysis (*P* < 0.0001).

In an effort to explore the relationship between p62 expression and breast cancer metastatic capacity, we first collected five pairs of clinical metastatic breast cancer and adjacent normal tissues, and conducted western blot analysis of p62 expression levels. Compared with adjacent normal tissues, p62 protein levels were up-regulated at varying degrees in all metastatic breast cancer tissues (Figure 1A). Consistently, analysis of gene expression value from The Cancer Genome Atlas (TCGA) showed that p62 mRNA expression levels in normal tissues (*n* = 110) were significantly lower than that in both primary tumour (*n* = 1065; −0.4355 versus −0.1485) and metastatic tumour tissues (*n* = 7; −0.4355 versus −0.1423) (Figure 1B). Further, the Kaplan–Meier survival analysis of 87 breast carcinoma specimens revealed a correlation between the higher p62 expression and reduced metastasis-free survival times (*P* = 0.011, GSE6532 from the GEO database) (Figure 1C). Moreover, 104 breast cancer samples from GEO database (GSE42568) by univariate analysis showed that high p62 expression was associated with decreased relapse-free survival times (*P* < 0.0001) (Figure 1D). These data suggest that p62 is accumulated in metastatic breast cancer and its overexpression correlates with poor clinical outcome of breast cancer.

### p62 promotes invasive phenotypes of breast cancer cells in vitro

**Figure 2.**
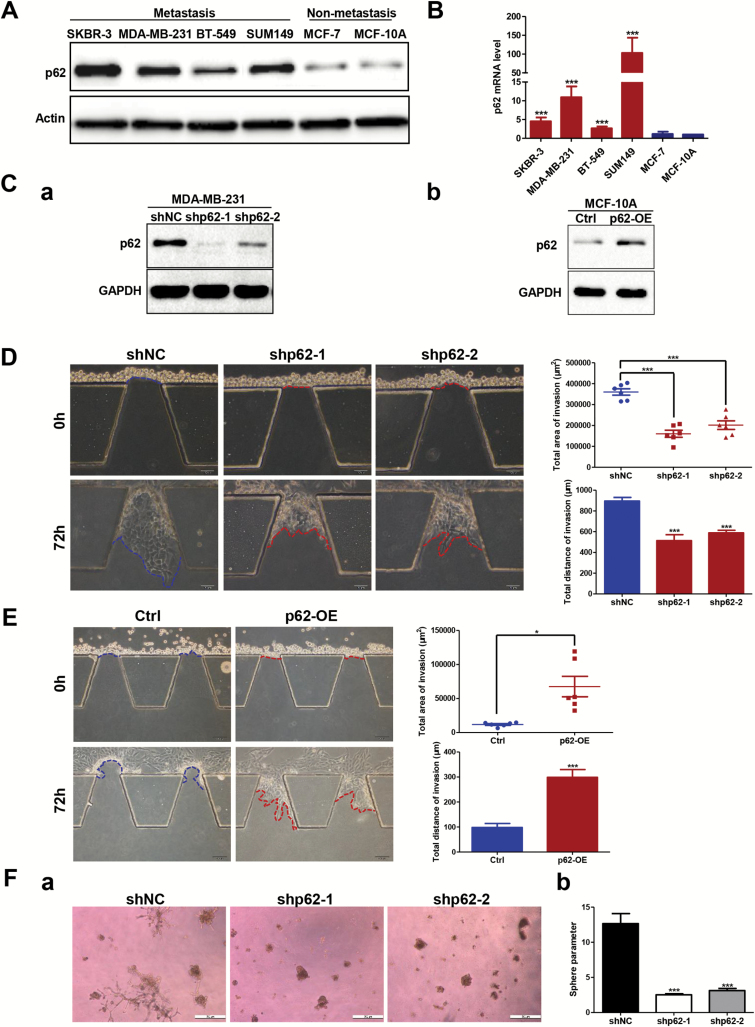
p62 is essential for breast cancer cells to maintain the invasive ability *in vitro*. (**A**) p62 protein expression was tested by western blot and (**B**) p62 mRNA expression was examined by RT-qPCR assay in both the metastatic and non-metastatic breast cancer cell lines. ***P < 0.001, two-tailed Student’s *t*-tests. Error bars represented mean ± standard deviation (SD). (**C**) Efficiencies for knockdown or overexpression of p62 were tested by western blot analysis. (**D**) Comparison of the invasive capacity in the microfluidic model by silencing p62 expression in MDA-MB-231 cells and (**E**) overexpressing p62 in MCF-10A cells. Scale bar, 100 μm. *P < 0.05, ***P < 0.001, two-tailed Student’s *t*-tests. Error bars represented mean ± SD. (**F-a**) Representative images of MDA-MB-231 control versus p62 knockdown cells embedded in three-dimensional Matrigel culture. Scale bar, 500 μm. (**F-b**) Quantification of tumour-sphere perimeter. ***P < 0.001, two-tailed Student’s *t*-tests. Error bars represented mean ± SD.

To investigate whether the expression level of p62 regulated invasive phenotypes of breast cancer cells, we first examined the expression of p62 in five breast cancer cell lines (SKBR-3, MDA-MB-231, BT-549, SUM149 and MCF-7), and a non-cancerous breast epithelial cell line (MCF-10A). Here, we found that both the mRNA and protein levels of p62 in metastatic group (SKBR-3, MDA-MB-231, BT-549 and SUM149) were much higher than that in non-metastatic group (MCF-7 and MCF-10A), indicating that the expression level of p62 positively correlated with the invasive phenotypes of breast cancer (Figure 2A and B). Then we used shRNA to mediate p62 depletion in metastatic MDA-MB-231 cells and lentivirus to facilitate ectopic overexpression of p62 in non-metastatic MCF-10A cells. The efficiencies of p62 knockdown and overexpression were assessed by western blotting. As shown in Figure 2C, p62 was effectively suppressed by two different p62 shRNA species in MDA-MB-231 cells, and significantly increased by lentivirus-infection in MCF-10A cells.

Herein, we built up a microfluidic model to observe local invasion of cancer cells in real time (Supplementary Figure 1 is available at *Carcinogenesis* Online) to assess the contribution of p62 in promoting breast cancer invasive ability. Silencing p62 expression dramatically reduced the invasive capacity of MDA-MB-231 cells as indicated by a decrease in both the area and distance of invasion (Figure 2D). Consistently, depletion of p62 impaired the ability of metastatic MDA-MB-231 cells to invade through matrigel pre-coated transwells (Supplementary Figure 2A is available at *Carcinogenesis* Online). In contrast, overexpressing p62 in non-metastatic MCF-10A cells resulted in the acquisition of invasive ability as indicated by both the microfluidic and Transwell invasion assays (Figure 2E; Supplementary Figure 2B is available at *Carcinogenesis* Online). Moreover, we examined the effect of p62 suppression on the ability of MDA-MB-231 cells to form colonies in three-dimensional matrigel culture. After 10 days in culture, control cells formed multiple protrusions invading into the surrounding matrix while MDA-MB-231 cells with p62 depletion formed smooth edges around cell spheres (Figure 2F-a). These results were quantified by calculation of the perimeter, which showed a significant difference between control and p62 knockdown cells (Figure 2F-b). Together, these observations indicate that p62 enhances breast cancer cells invasion.

### Inhibition of p62 attenuates breast cancer metastasis and leads to decreased tumourigenicity in vivo

**Figure 3.**
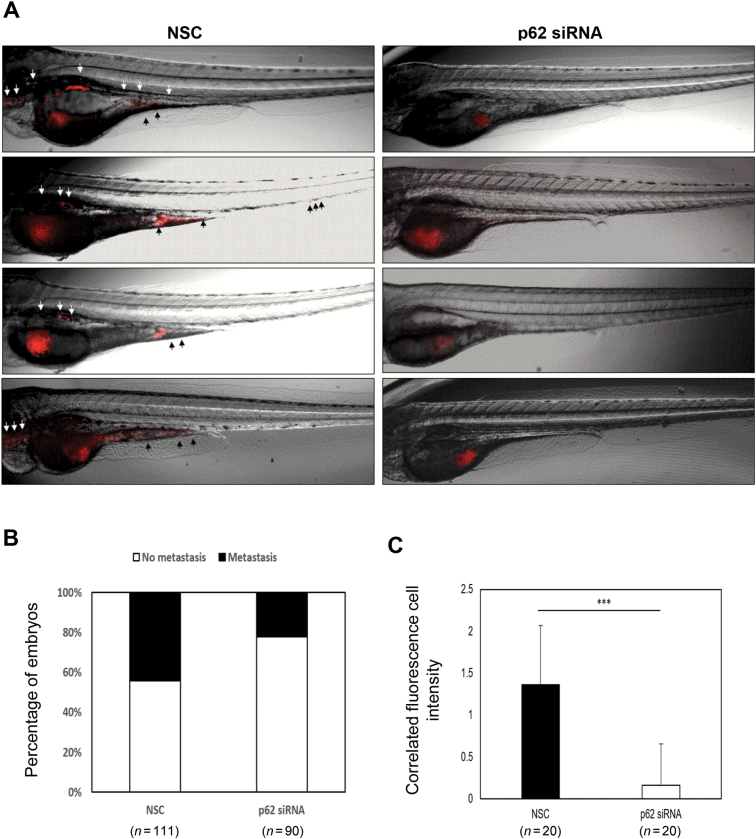
Effects of knockdown p62 on cell migration in Tra/Nac zebrafish embryos. MCF-7 cells were transfected with non-specific control or p62 siRNA, respectively. Thirty embryos were injected for each group and approximately 150 CM-Dil labelled MCF-7 cells were injected into each 2 dpf Tra/Nac zebrafish embryos. Metastasis was measured under the fluorescent microscope at 3 days post-implantation. Some of the embryos were died at this stage. (**A**) Tra/Nac zebrafish embryos at 2 dpf transplanted with MCF-7 non-specific control and MCF-7 p62 siRNA. The implanted tumour cells can be seen in red (red fluorescent microscope). Presence of migrated tumour cells from yolk sac to the tail is indicated with a black arrow. (**B**) Percentage of zebrafish which presented any form of metastasis in the total number of injected zebrafish in the experiment. (**C**) The corrected total cell fluorescence (CTCF) was measured using the formula: CTCF= integrated density − (area of selected cell × mean fluorescence of background readings) for each imaged zebrafish. Scale bar, 500 µm. **P < 0.01, two-tailed Student’s *t*-tests. Error bars represented mean ± standard deviation.

**Figure 4.**
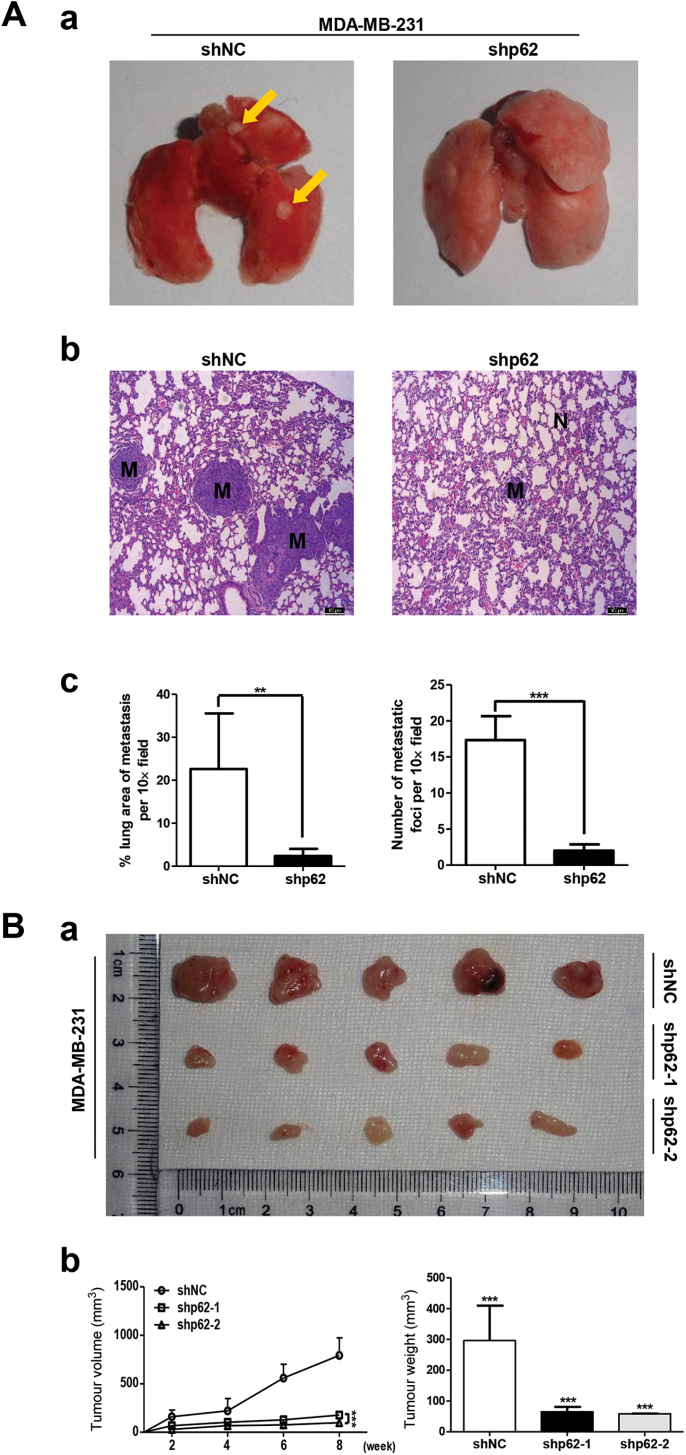
Inhibition of p62 attenuates breast cancer metastasis and leads to decreased tumourigenicity *in vivo*. (**A**) Control shRNA MDA-MB-231 and p62 shRNA MDA-MB-231 cells were injected into the lateral tail vein of 4–6 weeks old BALB/C (nu/nu) female nude mice. Macrograph of representative lungs (**A-a**) and haematoxylin and eosin staining of lung sections (**A-b**) were presented to show metastases in lungs. The metastatic nodules were indicated with yellow arrows. Scale bar, 100 μm. The area and number of metastatic nodules were presented as mean ± standard deviation (SD) (*n* = 6) (**A-c**). (**B**) Immunodeficient mice were subcutaneously inoculated with equal number of control and p62 knockdown MDA-MB-231 cells (1 × 10^6^ cells per mouse, *n* = 5). Photographs of tumours (**B-a**), tumour volumes and tumour weights were shown (**B-b**). ***P < 0.001, two-tailed Student’s *t*-tests. Error bars represented mean ± SD.

We next evaluated the *in vivo* effects of p62 depletion on breast cancer metastasis. In zebrafish embryo xenograft assay, we engrafted MCF-7 siNC and MCF-7 sip62 cells, stably expressing red fluorescent proteins, in a zebrafish embryo host. At 3 days post-implantation, silencing p62 expression in MCF-7 cells exhibited an obviously reduced invasive behaviour when compared to control cells (Figure 3A). In addition, zebrafish embryo engrafted with p62 siRNA MCF-7 cells had a lower metastases incidence (20/90, 22.2%) compared to control cells (49/111, 44.1%) (Figure 3B). Quantification of the fluorescence intensity of invasive cells showed a significant decrease elicited by p62 knockdown (Figure 3C). In mice xenograft models, control shRNA MDA-MB-231 and p62 shRNA MDA-MB-231 cells were injected into the lateral tail vein of 4–6 weeks old BALB/C (nu/nu) female nude mice. Eight weeks after injection, mice injected with MDA-MB-231-shp62 cells had a markedly decreased metastatic burden as measured by macrography and haematoxylin and eosin staining (Figure 4A-a and A-b). Consistently, the area and number of metastatic nodules in the lung of mice injected with MDA-MB-231-shp62 cells were significantly reduced as compared with that injected with MDA-MB-231-shNC cells (Figure 4A-c). These results indicate that silencing p62 expression attenuates the metastatic ability of breast cancer.

To determine the effect of p62 knockdown on tumour growth, equal number of control and p62 knockdown MDA-MB-231 cells (1 × 10^6^ cells) were subcutaneously injected into 4–6 weeks old BALB/C (nu/nu) female nude mice (five mice in each group). Tumour xenografts were then measured every week and mice sacrificed after 8 weeks. As shown in Figure 4B, comparing with mice inoculated with MDA-MB-231-shNC cells, which formed large tumours within 56 days, the mice inoculated with MDA-MB-231-shp62-1 and shp62-2 cells showed a significant reduction in the tumour growth as indicated by the decreased tumour volumes and tumour weights. These results demonstrate that p62 suppression contributes to decreased tumourigenicity in breast cancer.

### Identification of vimentin as a novel p62 binding partner and p62 suppression downregulates vimentin protein expression

**Figure 5.**
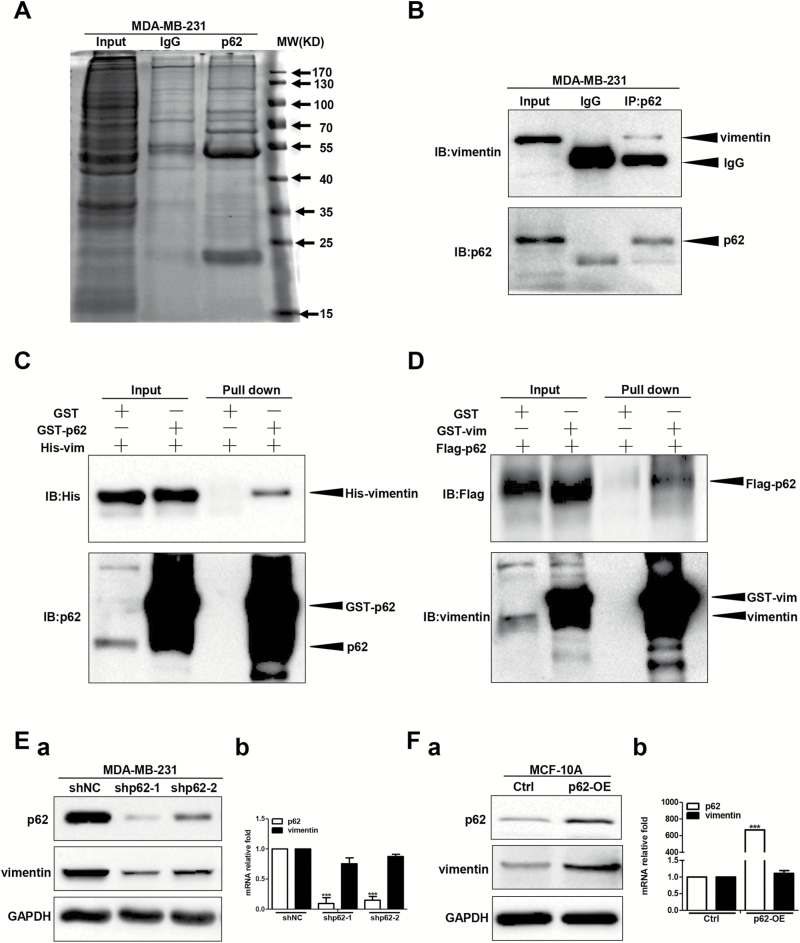
p62 binds to vimentin and p62 suppression downregulates vimentin protein expression. (**A**) The endogenous p62 co-immunoprecipitated proteins were analysed by mass spectrometry. (**B**) The interaction between endogenous p62 and vimentin protein was analysed by co-immunoprecipitation in MDA-MB-231 cells. (**C**, **D**) Protein interaction was analysed by glutathione S-transferase pull-down assay. Vimentin protein and mRNA levels were examined in p62 knockdown MDA-MB-231 cells (**E**) or p62 overexpression MCF-10A cells (**F**). The protein and mRNA levels of p62 and vimentin were tested by western blot and RT-qPCR assay, respectively. ***P < 0.001, two-tailed Student’s *t*-tests. Error bars represented mean ± standard deviation.

To further explore the mechanism for p62-mediated breast cancer invasion and metastasis, we sought to identify novel p62 binding proteins. Taking advantage of the proteomics-based approach, we analysed endogenous p62 co-immunoprecipitated proteins by mass spectrometry to identify putative p62 protein binding partners (Figure 5A; Supplementary Tables 2 and 3 are available at *Carcinogenesis* Online). Here, we focused our investigations on vimentin, which has been revealed to be a major intermediate filament in mediating cell movement (27,37,38). To this end, we first examined whether endogenous p62 bound to vimentin by co-immunoprecipitation in MDA-MB-231 cells. In these cell extracts, we detected vimentin after immunoprecipitation with a p62 antibody, but not with the control IgG, indicating that p62 interacted with vimentin (Figure 5B). In addition, the p62–vimentin interaction was further confirmed by glutathione S-transferase pull-down assay, by which GST-tagged p62 co-precipitated with His-tagged vimentin, as well as GST-tagged vimentin cooperated with Flag-tagged p62 (Figure 5C and D). Furthermore, immunofluorescence studies using p62 and vimentin specific antibodies showed that both proteins co-localized in the cytoplasm in MDA-MB-231 cells (Supplementary Figure 3 is available at *Carcinogenesis* Online). Together, these data suggest that p62 binds to vimentin in breast cancer cells.

Next, we examined the effects of silencing or overexpressing p62 on both the mRNA and protein levels of endogenous vimentin. As shown in Figure 5E-a and Supplementary Figure 4A-a, available at *Carcinogenesis* Online, shRNA or RNAi mediated depletion of p62 downregulated the protein expression levels of vimentin in both MDA-MB-231 and SK-BR-3 cells. In contrast, lentivirus-mediated overexpression of p62 upregulated vimentin protein levels in both MCF-10A and MCF-7 cells (Figure 5F-a; Supplementary Figure 4B-a is available at *Carcinogenesis* Online). Interestingly, vimentin mRNA levels exhibited no significant differences whether in p62 knockdown MDA-MB-231 and SK-BR-3 cells or in p62 overexpression MCF-10A and MCF-7 cells compared with their relative controls (Figure 5E-b and F-b; Supplementary Figure 4A-b and B-b is available at *Carcinogenesis* Online), suggesting that p62 promotes vimentin expression not at the transcriptional level.

### Vimentin plays an important role in p62-mediated breast cancer cells invasion

**Figure 6.**
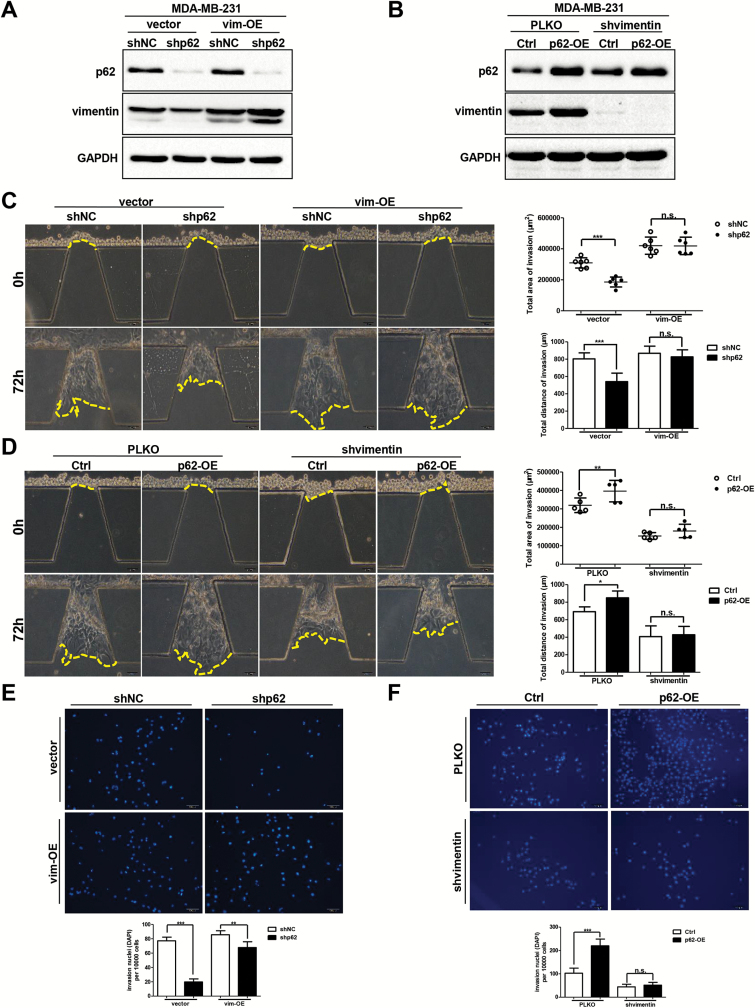
Vimentin is required for p62-mediated breast cancer cells invasion. p62 and vimentin expression levels were assessed by western blot assay in vimentin overexpressed control and p62 knockdown cells (**A**), or in shRNA-mediated vimentin suppressed control and p62 upregulation cells (**B**). The invasive ability was analysed by both the microfluidic assay (**C** and **D**) and the transwell invasion assay (**E** and **F**). Scale bar, 100 μm. *P < 0.05, **P < 0.01, ***P < 0.001, two-tailed Student’s *t*-tests. Error bars represented mean ± standard deviation.

In order to test if vimentin acted as an important mediator in p62 promoting breast cancer cells invasion, we overexpressed vimentin in both MDA-MB-231-shNC and MDA-MB-231-shp62 cells. Western blot assays were conducted to evaluate the protein levels of p62 and vimentin. As shown in Figure 6A, depletion of p62 attenuated vimentin expression, but overexpression of vimentin increased vimentin protein levels in both shNC and shp62 cells. Next, we performed the microfluidic assay to verify the effect of vimentin on p62-mediated cancer cells invasion. In accordance with previous reports, overexpressing vimentin resulted in a significant increment in the invasive ability of MDA-MB-231 cells (Figure 6C). Critically, re-constitution of vimentin expression in MDA-MB-231-shp62 cells abolished the reduction in invasive capacity (Figure 6C). Transwell invasion assay showed that both the vimentin overexpressing shNC cells and shp62 cells exhibited an increased invasive ability when compared to their relative shNC and shp62 cells, and the difference in the invasive ability of vimentin overexpressed shNC and shp62 cells was not so dramatically as the difference in shNC and shp62 cells (Figure 6E).

In addition, we conducted shRNA-mediated vimentin suppression in both MDA-MB-231-Ctrl and MDA-MB-231-p62-OE cells. Notably, overexpressing p62 caused an increment in vimentin protein expression in MDA-MB-231 cells, along with an increased invasive phenotype as indicated by both the microfluidic and Transwell assays (Figure 6B, D and F). Interestingly, knockdown of vimentin in MDA-MB-231-p62-OE cells attenuated the increased invasive ability and exhibited no significant difference in the capacity of invasion compared with vimentin knockdown MDA-MB-231-Ctrl cells (Figure 6D and F). Taken together, these results demonstrate that vimentin plays an essential role in p62-mediated invasion in breast cancer cells.

### p62 expression is positively correlated with vimentin protein levels in clinical breast cancer specimens

**Figure 7.**
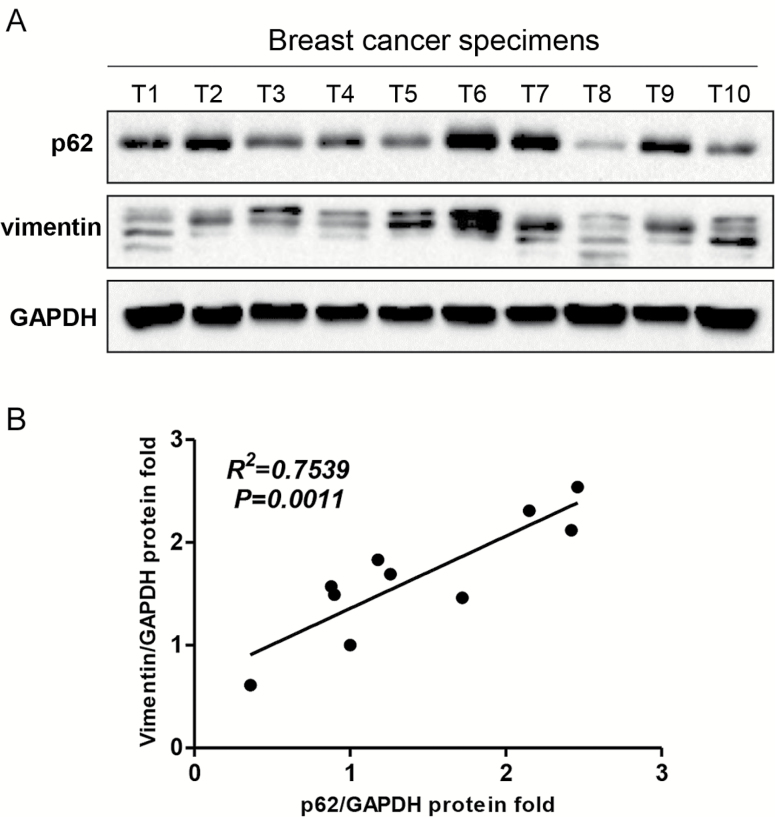
p62 expression is positively correlated with vimentin level in breast cancer specimens. (**A**) Both the p62 and vimentin protein expression in breast cancer specimens (*n* = 10; T: tumour) were subjected to western blot analysis. (**B**) Both the p62 and vimentin expression levels were normalized to relative glyceraldehyde 3-phosphate dehydrogenase and linear regression analysis was shown. *R*^2^ = 0.7539, *P* = 0.0011.

Finally, we assessed p62 and vimentin protein expression levels in clinical breast cancer specimens by western blot analysis. The expression of vimentin was relatively higher in samples which showed much more abundant p62 expression (Figure 7A). Linear regression analysis showed a positive correlation between p62 and vimentin protein expression (*R*^2^ = 0.7539, *P* = 0.0011) (Figure 7B), indicating that p62 overexpression is associated with high vimentin levels in clinical breast cancer tissues.

## Discussion

Our recent work demonstrated that the signalling adaptor p62 enhances breast cancer stem-like properties through downregulating let7a/b expression to stabilize MYC mRNA (39). In the present study, we further focus on the role of p62 in regulation of breast cancer metastasis. Several novel findings have been observed, and they include (i) p62 expression is elevated in metastatic breast cancer and its overexpression correlates with poor prognosis (Figure 1); (ii) p62 promotes invasive phenotypes of breast cancer cells *in vitro* and inhibition of p62 attenuates breast cancer metastasis and leads to decreased tumourigenicity *in vivo* (Figures 2–4; Supplementary Figure 2 is available at *Carcinogenesis* Online); (iii) p62 binds to vimentin and p62 suppression downregulates vimentin protein expression (Figure 5; Supplementary Figures 3 and 4 are available at *Carcinogenesis* Online); (iv) vimentin is required for p62-mediated breast cancer cells invasion (Figure 6); (v) p62 expression is positively correlated with vimentin protein levels in clinical breast cancer specimens (Figure 7).

Considering that metastases are responsible for >90% of breast cancer-related deaths and that no available strategies to interfere with the progress effectively, the verification of reliable prognostic biomarkers and novel therapeutic targets underlying tumour metastasis is urgently needed. We report here for the first time that clinical metastatic breast cancer tissues display high levels of p62 expression (Figure 1). Further analysis confirmed that p62 is required for the maintenance of breast cancer invasion and metastasis (Figures 2–4; Supplementary Figure 2 is available at *Carcinogenesis* Online). Notably, the metastases incidence of zebrafish embryo engrafted with MCF-7 control cells was 44.1% (49/111), whereas MCF-7 cells with p62 depletion had a lower incidence of 22.2% (20/90) within the same time frame (Figure 3B). Silencing p62 expression attenuates breast cancer cells metastasis in both zebrafish embryo and mouse tail vein xenograft models (Figures 3C and 4A). Collectively, our data strongly suggest p62 as a potential target for limiting breast cancer metastasis.

Interestingly, a recent study showed that intramuscularly administered p62-encoding plasmid induces anti-p62 antibodies and exhibits strong antitumour activity in models of allogeneic mouse tumours, including the Ca755 breast carcinoma (40). p62 DNA vaccine also decreases or stabilizes growth of locally advanced mammary tumours in dogs (41). These two studies indicate that p62 might be a potential candidate antigen for cancer immunotherapy. A feasible cancer antigen should be immunogenic, essential for cancer cells but not for normal tissues to reduce the risk of toxicity, and overexpressed in tumours as compared to the normal tissues (42). Here, our data show that p62 is overexpressed in breast cancer tissues relative to adjacent normal breast tissues (Figure 1A). High p62 expression levels correlate with reduced metastasis- and relapse-free survival times (Figure 1C and D). In agreement, previous studies also implied that p62 is accumulated in Ras-transformed cells and it is required for Ras-induced survival and transformation (43). Moreover, we identified a critical role of p62 in the maintenance of breast cancer stemness (39). In combination, our observations reveal the dependence of breast tumours on p62 in contrast to normal breast tissues, further expanding the suggestion that p62 protein might be an excellent target as a cancer antigen.

The scaffold protein p62 includes several important functional domains, which mediate the interaction with different signalling molecules to regulate cancer initiation and progression (44,45). For example p62 has been reported to promote engrafted human skin cancer cell growth through interacting with Twist-1 and inhibiting Twist-1 autophagic degradation (46). p62 also specially associates with mTORC1 by binding to raptor to regulate cell growth and autophagy that integrates nutrient sensing and cell size control (10,47,48). In an effort to investigate the mechanisms underlying the regulation of breast cancer metastasis by p62, we found that p62 binds to vimentin and silencing p62 expression downregulates vimentin protein expression (Figure 5; Supplementary Figures 3 and 4 are available at *Carcinogenesis* Online). Most importantly, vimentin is required for p62-promoted breast cancer metastasis (Figure 6). Linear regression analysis of clinical breast cancer specimens indicated that p62 is positively correlated with vimentin protein expression (Figure 7), while in adjacent normal samples, there is no such obviously correlation between p62 and vimentin expression (Supplementary Figure 5 is available at *Carcinogenesis* Online). Still, the molecular mechanism whereby p62 regulates vimentin expression remains to be unknown. We hypothesized that p62 regulates vimentin protein expression through ubiquitin-proteasome pathway. In order to test this conjecture, we performed reciprocal immunoprecipitation assay and analysed proteins via immunoblotting. Ubiquitinated vimentin could be readily detected (Supplementary Figure 6A and B is available at *Carcinogenesis* Online). Then, we treated control and p62 knockdown MDA-MB-231 cells with MG132, a potent proteasome inhibitor, and found that the effect of p62 depletion mediated vimentin suppression was not reversed effectively (Supplementary Figure 6C is available at *Carcinogenesis* Online). Moreover, treatment with MG132 in MCF-10A-Ctrl and MCF-10A-p62-OE cells, failed to prevent the upregulation of vimentin expression levels by p62 overexpression (Supplementary Figure 6D is available at *Carcinogenesis* Online). Further, to confirm whether p62 upregulation influences the ubiquitin of vimentin, we overexpressed p62 in HEK-293T cells and used His-tagged vimentin to co-immunoprecipitate with Flag-tagged ubiquitin in the absence or presence of MG132. The IP/IB results showed that there were no significant differences in the vimentin ubiquitinated levels in both the control and p62 overexpressing cells (Supplementary Figure 6E is available at *Carcinogenesis* Online). Together these data indicate that depletion of p62 downregulates vimentin protein expression independent of the ubiquitin-proteasome pathway.

In summary, we demonstrate that the signalling adaptor p62 enhances breast cancer metastasis through interacting with vimentin. Our findings also suggest that p62 could be used as both the potential prognostic biomarker of metastatic breast cancer and the therapeutic target for metastatic breast cancer treatment.

## Supplementary Material

Supplementary data are available at *Carcinogenesis* online.

## Funding

This work is supported by the National Basic Research Program of China (973 Program; 2012CB967000 to Q.L.), National Natural Science Foundation of China (81573025 to Q.L., 81201686 to J.X., 81402445 to C.-L.W., 81602588 to L.-Z.X. and 81602585 to F.-M.Z.) and the Liaoning (NSF2014029102 to Q.L. and 201601231 to L.-Z.X.). Eric W.-F. Lam’s work is supported by CRUK (A12011), Breast Cancer Now (2012MayPR070; 2012NovPhD016) and Medical Research Council of the UK (MR/N012097/1).

## Supplementary Material

Figure-S1

Figure-S2

Figure-S3

Figure-S4

Figure-S5

Figure-S6

Supplementary-Table-1

Supplementary-Table-2

Supplementary-Table-3

Supplementary-Figure-Legends

## Abbreviations

CTCF: corrected total cell fluorescence
shRNA: short hairpin RNA

## References

[1] Valastyan S. (2011) Tumor metastasis: molecular insights and evolving paradigms. Cell., 147, 275–292. 22000009 10.1016/j.cell.2011.09.024 PMC3261217

[2] Fidler I.J (2003) The pathogenesis of cancer metastasis: the ‘seed and soil’ hypothesis revisited. Nat. Rev. Cancer., 3, 453–458. 12778135 10.1038/nrc1098

[3] Chambers A.F. (2002) Dissemination and growth of cancer cells in metastatic sites. Nat. Rev. Cancer., 2, 563–572. 12154349 10.1038/nrc865

[4] Gupta G.P. (2006) Cancer metastasis: building a framework. Cell., 127, 679–695. 17110329 10.1016/j.cell.2006.11.001

[5] Weigelt B. (2005) Breast cancer metastasis: markers and models. Nat. Rev. Cancer., 5, 591–602. 16056258 10.1038/nrc1670

[6] Yeo B. (2014) An update on the medical management of breast cancer. BMJ, 348, g3608. 24912480 10.1136/bmj.g3608

[7] Yokota J (2000) Tumor progression and metastasis. Carcinogenesis., 21, 497–503. 10688870 10.1093/carcin/21.3.497

[8] Lujambio A. (2009) How epigenetics can explain human metastasis: a new role for microRNAs. Cell Cycle., 8, 377–382. 19177007 10.4161/cc.8.3.7526

[9] Park I. (1995) Phosphotyrosine-independent binding of a 62-kDa protein to the src homology 2 (SH2) domain of p56lck and its regulation by phosphorylation of Ser-59 in the lck unique N-terminal region. Proc. Natl. Acad. Sci. U. S. A., 92, 12338–12342. 8618896 10.1073/pnas.92.26.12338 PMC40352

[10] Duran A. (2011) p62 is a key regulator of nutrient sensing in the mTORC1 pathway. Mol. Cell., 44, 134–146. 21981924 10.1016/j.molcel.2011.06.038 PMC3190169

[11] Jain A. (2010) p62/SQSTM1 is a target gene for transcription factor NRF2 and creates a positive feedback loop by inducing antioxidant response element-driven gene transcription. J. Biol. Chem., 285, 22576–22591. 20452972 10.1074/jbc.M110.118976 PMC2903417

[12] Kirkin V. (2009) A role for ubiquitin in selective autophagy. Mol. Cell., 34, 259–269. 19450525 10.1016/j.molcel.2009.04.026

[13] Pankiv S. (2007) p62/SQSTM1 binds directly to Atg8/LC3 to facilitate degradation of ubiquitinated protein aggregates by autophagy. J. Biol. Chem., 282, 24131–24145. 17580304 10.1074/jbc.M702824200

[14] Kitamura H. (2006) Cytosolic overexpression of p62 sequestosome 1 in neoplastic prostate tissue. Histopathology., 48, 157–161. 16405664 10.1111/j.1365-2559.2005.02313.x

[15] Komatsu M. (2010) The selective autophagy substrate p62 activates the stress responsive transcription factor Nrf2 through inactivation of Keap1. Nat. Cell Biol., 12, 213–223. 20173742 10.1038/ncb2021

[16] Ling J. (2012) KrasG12D-induced IKK2/β/NF-κB activation by IL-1α and p62 feedforward loops is required for development of pancreatic ductal adenocarcinoma. Cancer Cell., 21, 105–120. 22264792 10.1016/j.ccr.2011.12.006 PMC3360958

[17] Inoue D. (2012) Accumulation of p62/SQSTM1 is associated with poor prognosis in patients with lung adenocarcinoma. Cancer Sci., 103, 760–766. 22320446 10.1111/j.1349-7006.2012.02216.x PMC7659245

[18] Li L. (2013) SQSTM1 is a pathogenic target of 5q copy number gains in kidney cancer. Cancer Cell., 24, 738–750. 24332042 10.1016/j.ccr.2013.10.025 PMC3910168

[19] Liu J.L. (2014) Prognostic significance of p62/SQSTM1 subcellular localization and LC3B in oral squamous cell carcinoma. Br. J. Cancer., 111, 944–954. 24983366 10.1038/bjc.2014.355 PMC4150268

[20] Thompson H.G. (2003) p62 overexpression in breast tumors and regulation by prostate-derived Ets factor in breast cancer cells. Oncogene., 22, 2322–2333. 12700667 10.1038/sj.onc.1206325

[21] Rolland P. (2007) The ubiquitin-binding protein p62 is expressed in breast cancers showing features of aggressive disease. Endocr. Relat. Cancer., 14, 73–80. 17395976 10.1677/erc.1.01312

[22] Luo R.Z. (2013) Accumulation of p62 is associated with poor prognosis in patients with triple-negative breast cancer. Onco. Targets. Ther., 6, 883–888. 23888115 10.2147/OTT.S46222 PMC3722135

[23] Puvirajesinghe T.M. (2016) Identification of p62/SQSTM1 as a component of non-canonical Wnt VANGL2-JNK signalling in breast cancer. Nat. Commun., 7, 10318. 26754771 10.1038/ncomms10318 PMC4729931

[24] Yang J. (2008) Epithelial-mesenchymal transition: at the crossroads of development and tumor metastasis. Dev. Cell., 14, 818–829. 18539112 10.1016/j.devcel.2008.05.009

[25] Satelli A. (2011) Vimentin in cancer and its potential as a molecular target for cancer therapy. Cell. Mol. Life Sci., 68, 3033–3046. 21637948 10.1007/s00018-011-0735-1 PMC3162105

[26] Thiery J.P (2002) Epithelial-mesenchymal transitions in tumour progression. Nat. Rev. Cancer., 2, 442–454. 12189386 10.1038/nrc822

[27] Chernoivanenko I.S. (2013) Role of vimentin in cell migration. Ontogenez., 44, 186–202. 23885566 10.7868/s0475145013030026

[28] Wu M. (2007) Proteome analysis of human androgen-independent prostate cancer cell lines: variable metastatic potentials correlated with vimentin expression. Proteomics., 7, 1973–1983. 17566973 10.1002/pmic.200600643

[29] Sun B. (2008) Identification of metastasis-related proteins and their clinical relevance to triple-negative human breast cancer. Clin. Cancer Res., 14, 7050–7059. 18981002 10.1158/1078-0432.CCR-08-0520

[30] Wei J. (2008) Overexpression of vimentin contributes to prostate cancer invasion and metastasis via src regulation. Anticancer Res., 28, 327–334. 18383865

[31] Chen Y.R. (2006) Quantitative proteomic and genomic profiling reveals metastasis-related protein expression patterns in gastric cancer cells. J. Proteome Res., 5, 2727–2742. 17022644 10.1021/pr060212g

[32] Eckes B. (2000) Impaired wound healing in embryonic and adult mice lacking vimentin. J. Cell Sci., 113(Pt 13), 2455–2462. 10852824 10.1242/jcs.113.13.2455

[33] Eckes B. (1998) Impaired mechanical stability, migration and contractile capacity in vimentin-deficient fibroblasts. J. Cell Sci., 111(Pt 13), 1897–1907. 9625752 10.1242/jcs.111.13.1897

[34] Zheng F.M. (2014) A novel small molecule aurora kinase inhibitor attenuates breast tumor-initiating cells and overcomes drug resistance. Mol. Cancer Ther., 13, 1991–2003. 24899685 10.1158/1535-7163.MCT-13-1029

[35] Xu L.Z. (2014) Aurora kinase a suppresses metabolic stress-induced autophagic cell death by activating mTOR signaling in breast cancer cells. Oncotarget., 5, 7498–7511. 25115395 10.18632/oncotarget.2241 PMC4202139

[36] Debnath J. (2003) Morphogenesis and oncogenesis of MCF-10A mammary epithelial acini grown in three-dimensional basement membrane cultures. Methods., 30, 256–268. 12798140 10.1016/s1046-2023(03)00032-x

[37] Lowery J. (2015) Intermediate filaments play a pivotal role in regulating cell architecture and function. J. Biol. Chem., 290, 17145–17153. 25957409 10.1074/jbc.R115.640359 PMC4498054

[38] Chou Y.H. (2007) The motility and dynamic properties of intermediate filaments and their constituent proteins. Exp. Cell Res., 313, 2236–2243. 17498691 10.1016/j.yexcr.2007.04.008

[39] Xu L.Z. (2017) p62/SQSTM1 enhances breast cancer stem-like properties by stabilizing MYC mRNA. Oncogene., 36, 304–317. 27345399 10.1038/onc.2016.202 PMC5269535

[40] Venanzi F. (2013) Broad-spectrum anti-tumor and anti-metastatic DNA vaccine based on p62-encoding vector. Oncotarget., 4, 1829–1835. 24121124 10.18632/oncotarget.1397 PMC3858567

[41] Gabai V. (2014) Pilot study of p62 DNA vaccine in dogs with mammary tumors. Oncotarget., 5, 12803–12810. 25296974 10.18632/oncotarget.2516 PMC4350343

[42] Gabai V.L. (2014) Feasibility analysis of p62 (SQSTM1)-encoding DNA vaccine as a novel cancer immunotherapy. Int. Rev. Immunol., 33, 375–382. 25277339 10.3109/08830185.2014.954699 PMC4438419

[43] Duran A. (2008) The signaling adaptor p62 is an important NF-kappaB mediator in tumorigenesis. Cancer Cell., 13, 343–354. 18394557 10.1016/j.ccr.2008.02.001

[44] Moscat J. (2012) p62: a versatile multitasker takes on cancer. Trends Biochem. Sci., 37, 230–236. 22424619 10.1016/j.tibs.2012.02.008 PMC3531712

[45] Moscat J. (2009) p62 at the crossroads of autophagy, apoptosis, and cancer. Cell., 137, 1001–1004. 19524504 10.1016/j.cell.2009.05.023 PMC3971861

[46] Qiang L. (2014) Regulation of cell proliferation and migration by p62 through stabilization of Twist1. Proc. Natl. Acad. Sci. U. S. A., 111, 9241–9246. 24927592 10.1073/pnas.1322913111 PMC4078859

[47] Moscat J. (2011) Feedback on fat: p62-mTORC1-autophagy connections. Cell., 147, 724–727. 22078874 10.1016/j.cell.2011.10.021 PMC3290994

[48] Kim D.H. (2002) mTOR interacts with raptor to form a nutrient-sensitive complex that signals to the cell growth machinery. Cell., 110, 163–175. 12150925 10.1016/s0092-8674(02)00808-5

