# Correction to: p62/SQSTM1 interacts with vimentin to enhance breast cancer metastasis

**DOI:** 10.1093/carcin/bgaf042

**Published:** 2025-08-25

This is a **correction** to:

Si-Si Li, Ling-Zhi Xu, Wei Zhou, Shang Yao, Chun-Li Wang, Jiang-Long Xia, He-Fei Wang, Muhammad Kamran, Xiao-Yuan Xue, Lin Dong, Jing Wang, Xu-Dong Ding, Laura Bella, Laurence Bugeon, Jie Xu, Fei-Meng Zheng, Margaret J Dallman, Eric W F Lam, Quentin Liu, p62/SQSTM1 interacts with vimentin to enhance breast cancer metastasis, *Carcinogenesis*, Volume 38, Issue 11, November 2017, Pages 1092–1103, https://doi.org/10.1093/carcin/bgx099

After article publication, the authors notified the journal that the representative image of PLKO-p62-OE-72h group in Figure 6D was misplaced. In the original article, the authors used the correct raw images to make their statistical analysis. However, these raw images were shown with the misplaced representative image of PLKO-p62-OE-72h group in Figure 6D. The statistical analysis result (right panel of Figure 6D) is correct, and the authors’ correction in Figure 6D does not affect the interpretation or conclusions of this article.

**Figure:**
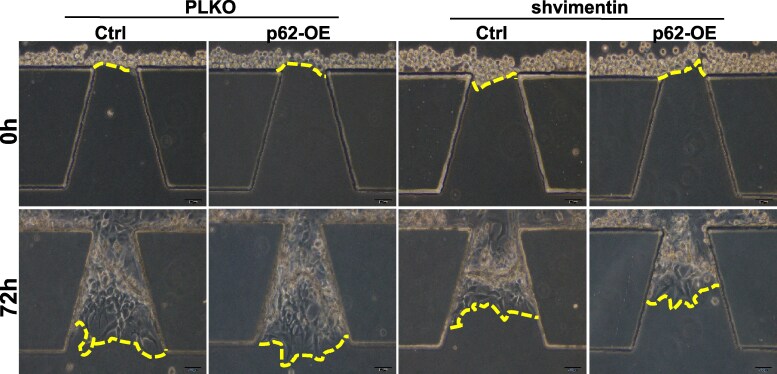


These details have been corrected only in this **correction notice** to preserve the published version of record.

