# Supplementary figures and images for: p62/SQSTM1 interacts with vimentin to enhance breast cancer metastasis
Source: Carcinogenesis. 2017 Sep 12;38(11):1092–103. doi: 10.1093/carcin/bgx099 (PMC5862327; doi:10.1093/carcin/bgx099)

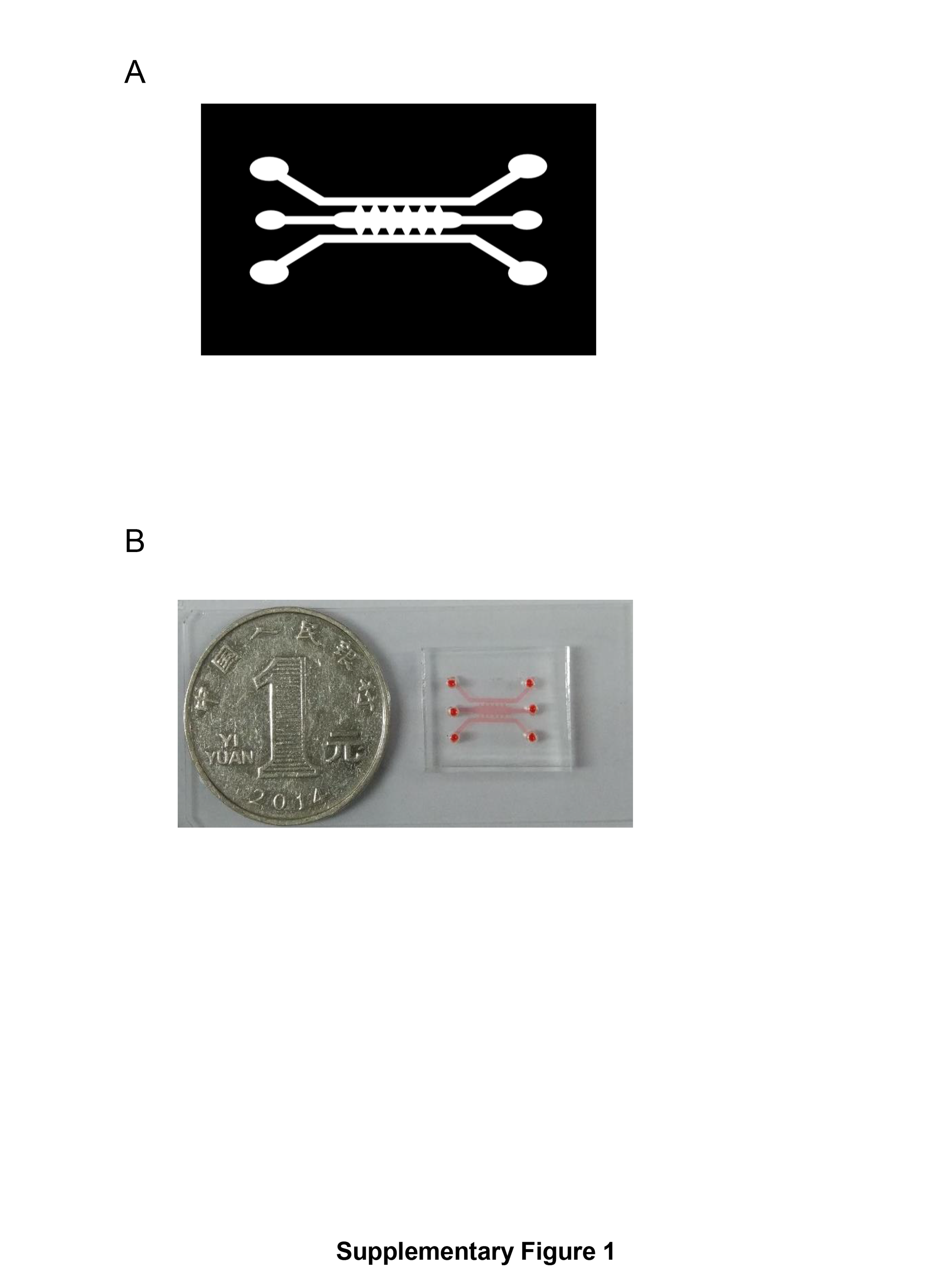

Supplement: Figure-S1 [file bgx099_suppl_figure-s1.png]

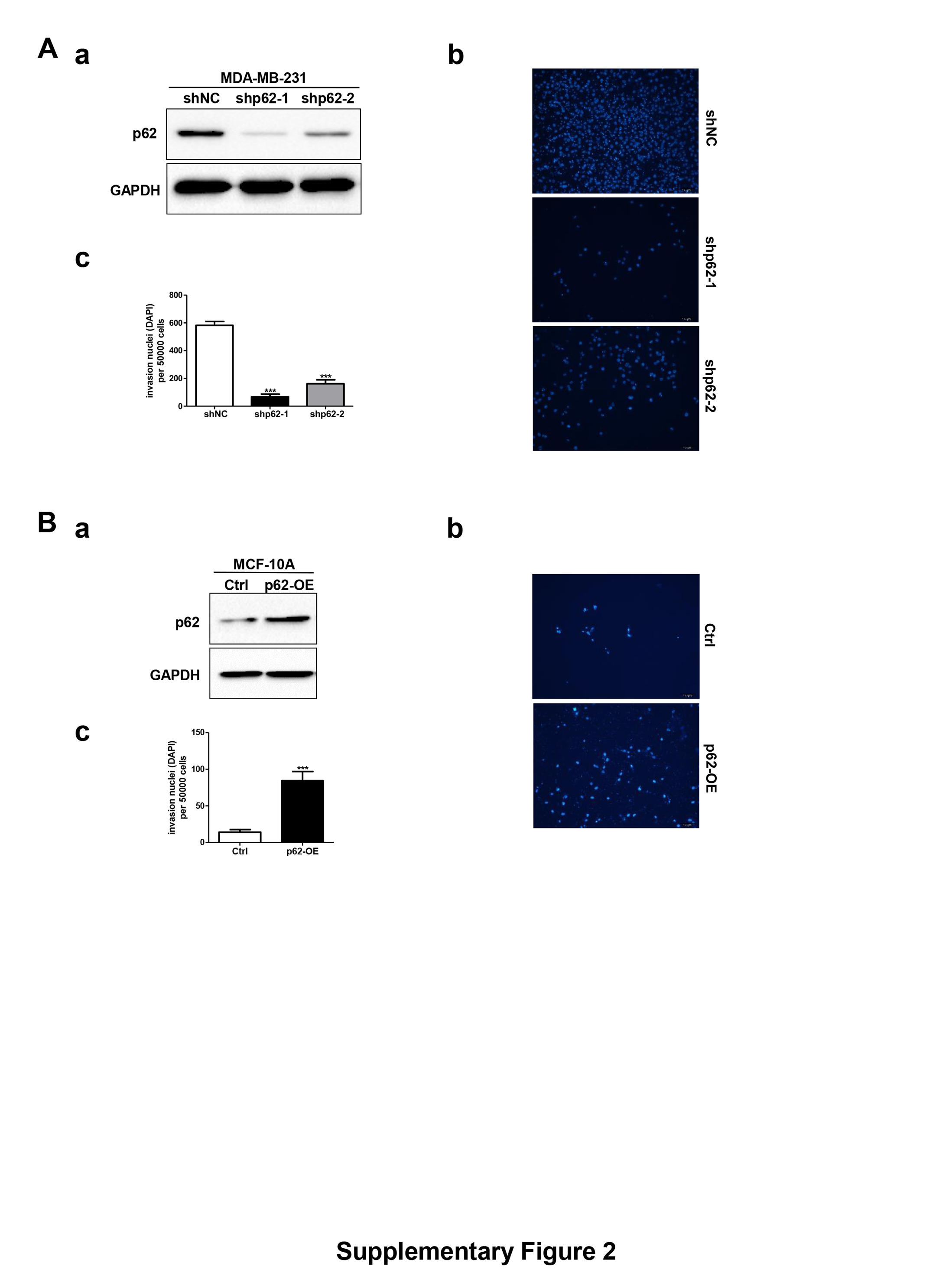

Supplement: Figure-S2 [file bgx099_suppl_figure-s2.png]

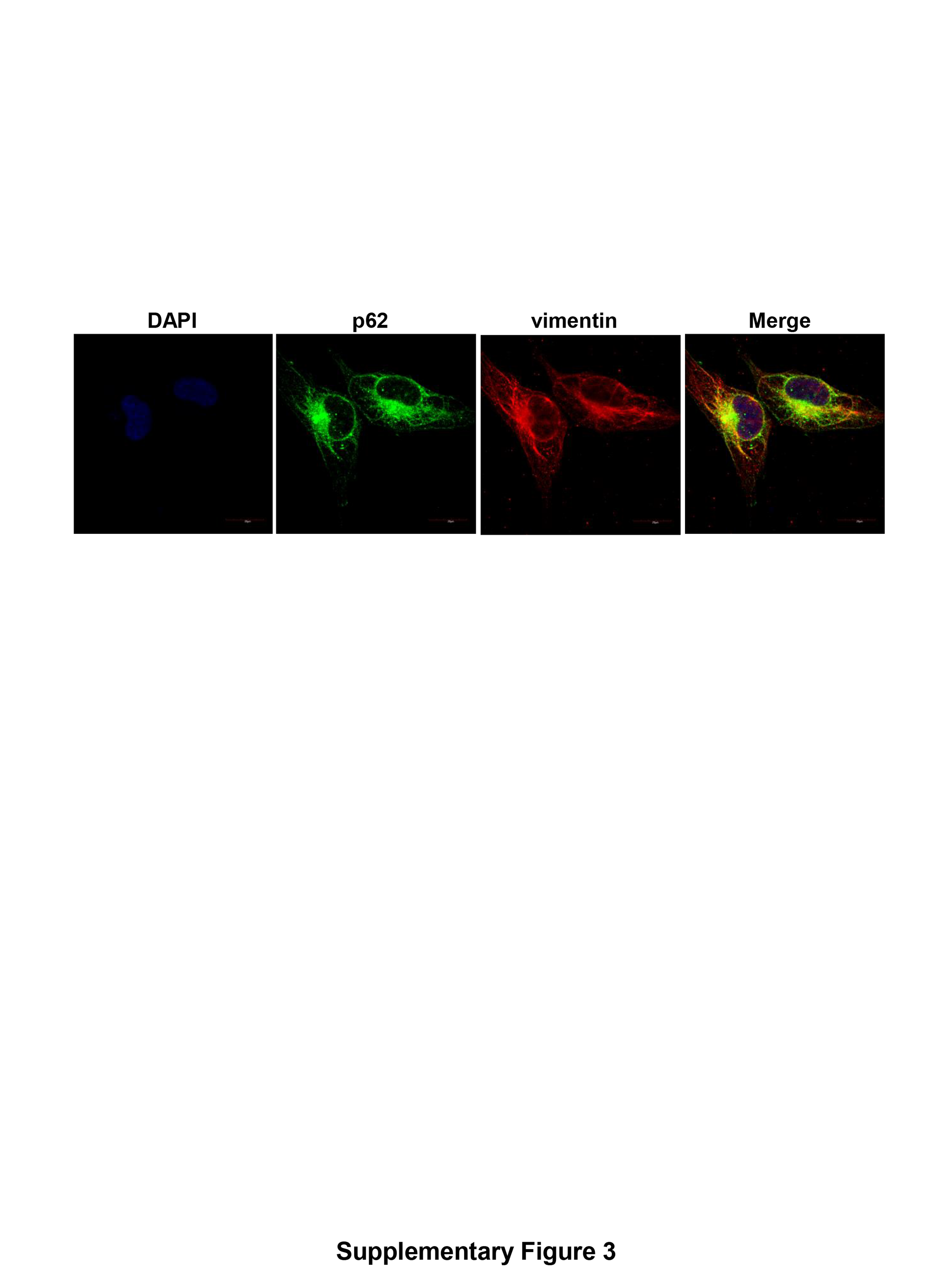

Supplement: Figure-S3 [file bgx099_suppl_figure-s3.png]

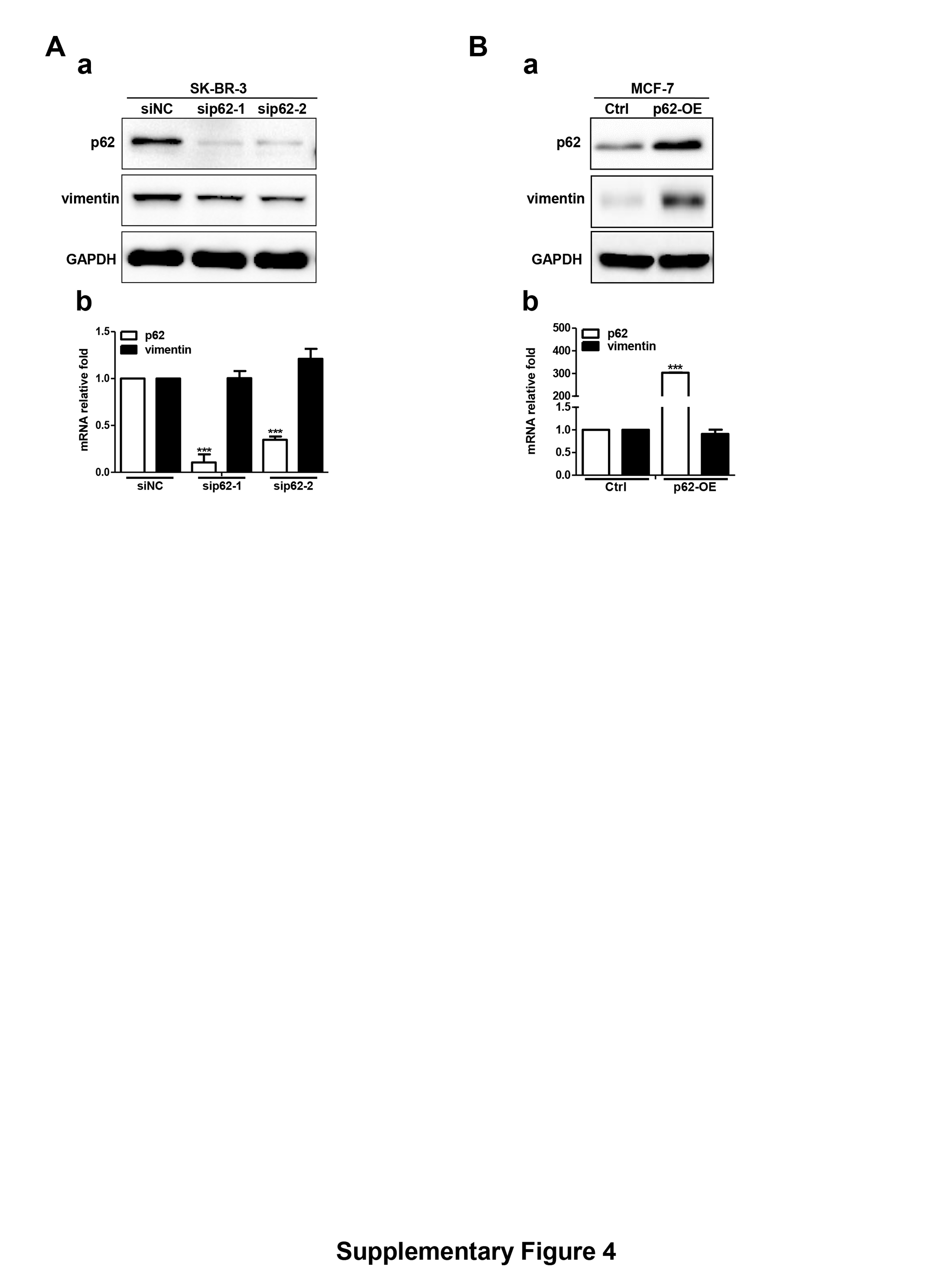

Supplement: Figure-S4 [file bgx099_suppl_figure-s4.png]

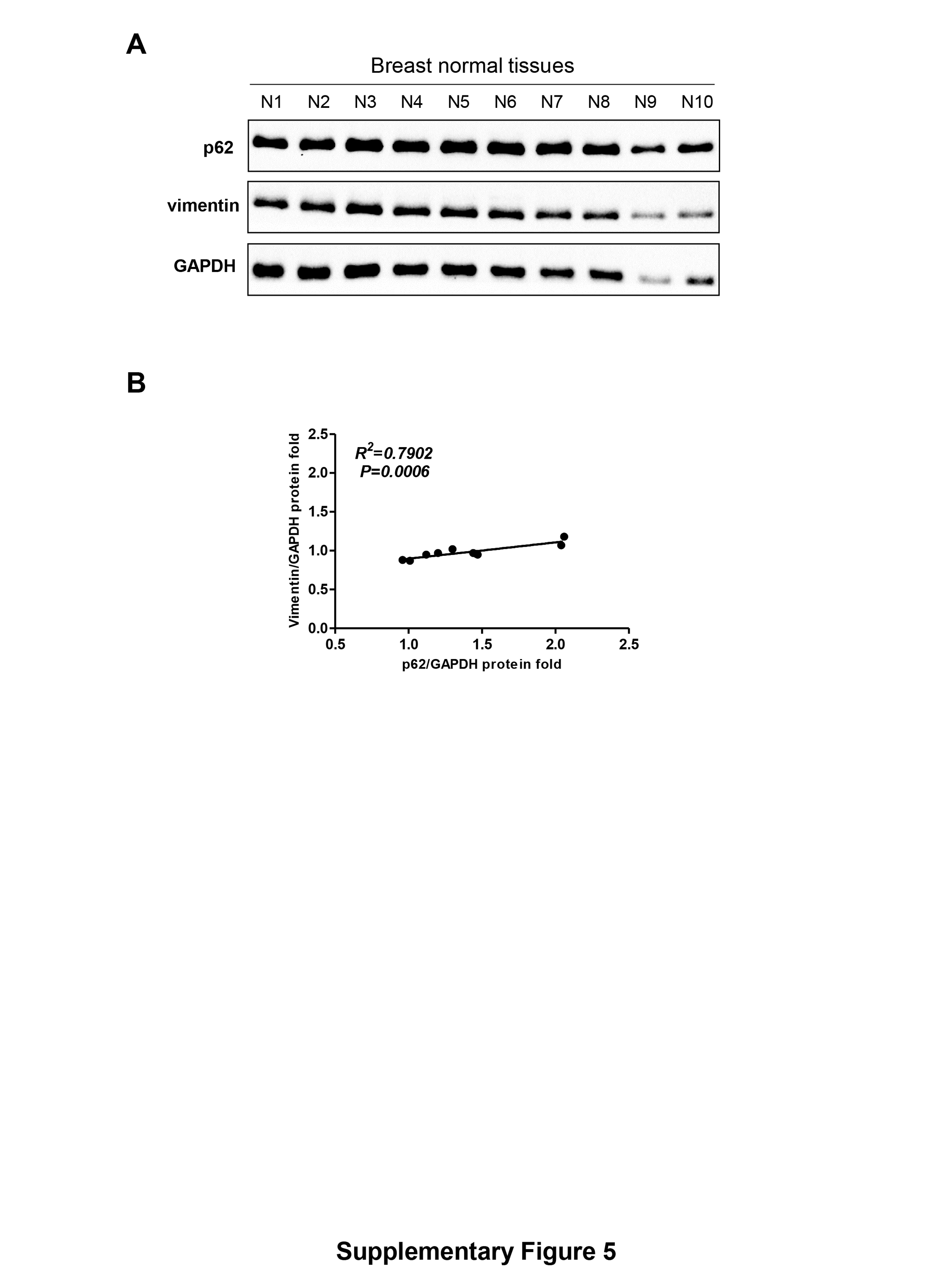

Supplement: Figure-S5 [file bgx099_suppl_figure-s5.png]

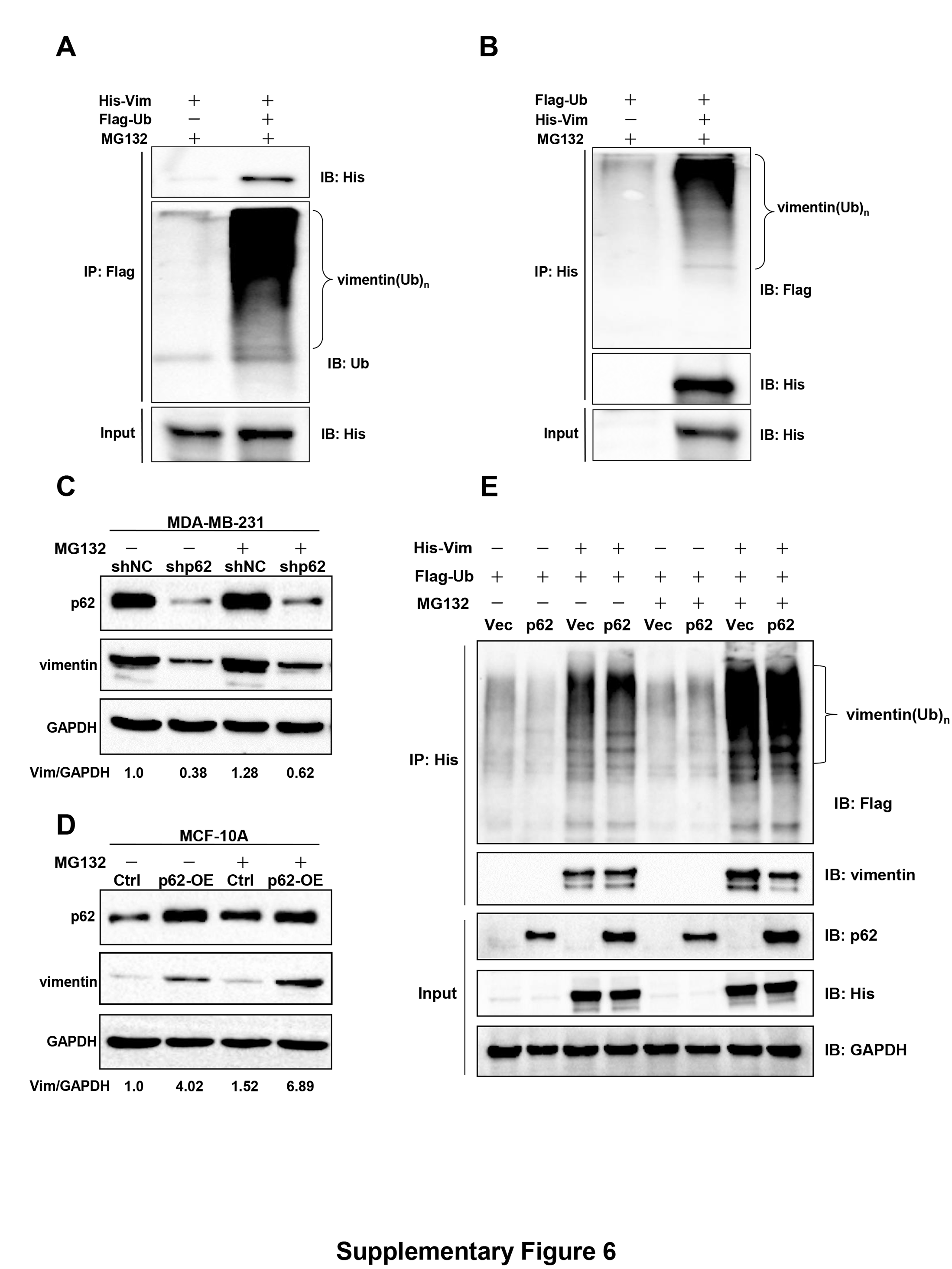

Supplement: Figure-S6 [file bgx099_suppl_figure-s6.png]
