# Supplementary material for: p62/SQSTM1 interacts with vimentin to enhance breast cancer metastasis
Source: Carcinogenesis. 2017 Sep 12;38(11):1092–103. doi: 10.1093/carcin/bgx099 (PMC5862327; doi:10.1093/carcin/bgx099)
Supplement: Supplementary-Figure-Legends [file bgx099_suppl_supplementary-figure-legends.docx]

**Supplementary Figure 1** (A) Model diagram and (B) photograph of the microfluidic chip for testing cancer cells local invasion abilities. The chip is composed of one central chamber and two side channels. Cancer cells in which contained 1% FBS cell culture medium are seeded in one of the side channels and the other side channel is injected into 10% FBS containing cell culture medium. Matrigel is mixed with same volume 10% FBS containing cell culture medium and is injected into the central channel.

**Supplementary Figure 2** The invasive properties of MDA-MB-231-shp62 (A) and MCF-10A-p62-OE cells (B) together with their relative controls were analyzed by Transwell invasion assay. ***P<0.001, two-tailed Student’s t-tests. Error bars represented mean ± SD.

**Supplementary Figure 3** Immunofluorescence staining of p62 (Green) and vimentin (Red) in MDA-MB-231 cells. Scale bars, 20 μm.

**Supplementary Figure 4** Vimentin protein and mRNA levels were examined in p62 knockdown SK-BR-3 cells or p62 overexpression MCF-7 cells. The protein and mRNA levels of p62 and vimentin were respectively tested by Western blot and RT-qPCR assay. ***P<0.001, two-tailed Student’s t-tests. Error bars represented mean ± SD.

**Supplementary Figure 5** (A) Both the p62 and vimentin protein expression in breast cancer adjacent normal tissues (n=10; N: normal) was subjected to Western blot analysis. (B) Both the p62 and vimentin expression levels were normalized to relative GAPDH and linear regression analysis was shown. R^2^=0.7902, P=0.0006.

**Supplementary Figure 6** (A) (B) HEK293T cells were co-transfected with His-Vimentin and Flag-Ubiquitin in the presence of MG132. Protein interaction was analyzed by co-immunoprecipitation and Western blot assay. (C) (D) The protein expression levels of p62 and vimentin were examined by Western blot assay. (E) Control and p62 overexpression HEK293T cells were co-transfected with Flag-Ubiquitin and His-Vimentin in the presence or absence of MG132. Vimentin ubiquitinated levels were analyzed by co-immunoprecipitation and Western blot assay.

**Supplementary Materials and Methods**

*Co-immunoprecipitation analysis*

Protein lysates (500-1000 μg) prepared from cultured cells. Immunocomplex pull-down was achieved via overnight incubation of protein lysates with relevant antibodies bound to Protein G-Agarose (Roche) or with Glutathione Sepharose beads (GE Healthcare) alone at 4℃. After careful washing, loading buffer was added, and the samples were boiled at 100℃ for 10 min. Co-immunoprecipitated proteins were then subjected to Western blot as described above.

*In-Gel Trypsin Digestion*

p62 and interacting proteins that were co-immunoprecipitated as described above were solubilized in 1.5 × SDS sample buffer (15% glycerol, 60 mM Tris [pH 6.8], 3% SDS, 7.5% ß-mercaptoethanol) and run on SDS-PAGE. After migration, the gels were fixed, and proteins were detected with Coomassie blue staining. p62 immunoprecipitated protein bands and IgG control group protein bands were cut out of the gels and rinsed three times in deionized water. Gel slices (≤ 1 mm) were incubated with 100 mM ammonium bicarbonate buffer for 15 min at 4℃. The supernatant was then removed, and samples were then mixed with 50% acetonitrile for shaking 10 min at 4℃ to dehydrate the gel pieces. Re-incubate with 100 mM ammonium bicarbonate buffer for 15 min at 4℃ and then mixed with 100% acetonitrile for shaking 10 min at 4℃. After dehydrating the gels, samples were incubated with DTT for 2 hours at 37℃. Following incubation, IAA was replaced with DTT and further incubated for 1 hour at 25℃ in darkness. Then samples were dealt with 50% acetonitrile, 50 mM ammonium bicarbonate and 100% acetonitrile sequentially. Finally, dried gels were covered with 500 μl trypsin solution (12.5 ng/μl in 25 mM ammonium bicarbonate) and in-gel digestion was performed at 37℃ overnight. The digested sample was prepared for mass spectrometry analysis as follows. Firstly, supernatant was transferred into a new tube and precipitants were incubated with 150 μl 25 mM ammonium bicarbonate for 10 min at 4℃, then put the supernatant into the same tube. Secondly, precipitants were added 250 μl buffer (ACN : TFA=94 : 6) for shaking 10 min at 4℃ and transferred into the same tube. Thirdly, dried the supernatant and store at -20℃. Finally, dissolved the samples in 0.1% FA totally and then prepared for mass spectrometric analysis.

*Mass Spectrometry*

Peptides were analyzed on LTQ XL mass spectrometer (Thermo, San Jose, CA) with 1D RPLC separating system in the positive ion mode. The control and experiment group were respectively loaded onto and separated by a C18 capillary column (Thermo, BioBasic-18, 150*0.1) with a 120 min RP gradient elution. A Finnigan surveyor MS pump (Thermo, San Jose, CA, USA) was used to deliver the mobile phase consisted of mobile A, 0.1% (v/v) formic acid in water, and mobile phase B, 0.1% (v/v) formic acid in ACN. The flow rate was adjusted to 300μL/min without splitting. The gradients elution was performed with gradients of 2−35% B in 90 min, 35−80% B in 2 min, 80% B in 10 min, 80% B−98% A in 3 min and 98% A in 20 min. All MS and MS/MS spectra were acquired in the data-dependent analysis mode, in which the 10 most intense ions with MS scan were selected for MS/MS scan by collision-induced dissociation (CID). All MS/MS spectra were searched using Proteome Discoverer (Version 1.3.0.339) against NCBI protein database (human20141121) with 1% false discovery rate (FDR). The parameters were set as follows: precursor-ion mass tolerance, 2 Da; fragment-ion mass tolerance, 1 Da; enzyme, trypsin (KR/P); missed cleavage, 2; static modification, Cys (+57.021 Da); dynamic modifications methionine (+15.995 Da). The results were shown in Supplementary Table 2 and 3.

*Immunofluorescence staining*

To determine the localization of p62 and vimentin, we performed immunofluorescence assay. In brief, cells were plated on coverslips to 30% confluence and then fixed in 4% para-formaldehyde-PBS at room temperature for 20 minutes and permeabilized in 0.5% Triton X-100 in PBS for 10 minutes at 4℃. Cells were then blocked with 1% BSA and incubated with primary antibody against both p62 (Santa Cruz, sc-28359) and vimentin (Cell Signaling Technology, 5741s), followed by a FITC conjugated second antibody (Invitrogen), counterstained with DAPI (1 μg/μl), and visualized using a confocal microscope (Leica).

*Statistical analysis*

The two-tailed Student’s t-tests was used to perform a statistical comparison between two groups. Statistical tests were performed using the SPSS software, version 16.0 (SPSS Inc.) or with GraphPad Prism 5.0 (GraphPad Software, Inc.). The level of statistical significance was set at *P < 0.05, **P <0.01, ***P <0.001.
